# Toward Methanol Production by CO_2_ Hydrogenation beyond Formic Acid Formation

**DOI:** 10.1021/acs.accounts.4c00411

**Published:** 2024-09-16

**Authors:** Naoya Onishi, Yuichiro Himeda

**Affiliations:** † National Institute of Advanced Industrial Science and Technology, 16-1 Onogawa, Tsukuba, Ibaraki 305-8569, Japan; ‡ Faculty of Pure and Applied Sciences, University of Tsukuba, Tsukuba, 1-1-1 Tennodai, Tsukuba, Ibaraki 305-8573, Japan

## Abstract

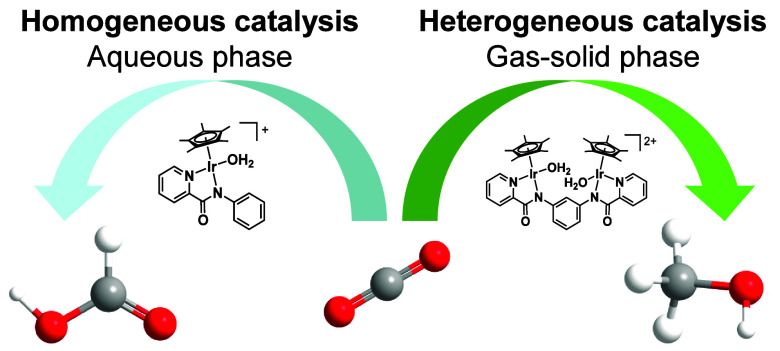

The Paradigm shift in considering CO_2_ as an alternative carbon feedstock as opposed to a waste product has recently prompted intense research activities. The implementation of CO_2_ utilization may be achieved by designing highly efficient catalysts, exploring processes that minimize energy consumption and simplifying product purification and separation. Among possible target products derived from CO_2_, methanol is highly valuable because it can be used in various chemical feedstocks and as a fuel. Although it is currently produced on a plant scale by heterogeneous catalysis using a Cu/ZnO-based catalyst, a limited theoretical conversion ratio at high reaction temperatures remains an issue. In addition, a catalytic system that can be adjusted to accommodate a variable renewable energy source for the synthesis of methanol is more desirable than current continuous-operation systems, which require a reliable energy supply. Recently, significant progress has been made in the field of homogeneous catalysis, which primarily relies on an indirect route to synthesize methanol via the hydrogenation of carbonate or formate derivatives in the presence of additives and solvents. However, homogeneous catalysis is inappropriate for industrial-scale methanol production because of the inefficient separation and purification processes involved.

In this Account, we demonstrate a novel approach for methanol production under mild reaction conditions by CO_2_ hydrogenation catalyzed by multinuclear iridium complexes under heterogeneous gas–solid phase conditions without any additives and solvents. One of the aims of this Account provides insights for overcoming the barriers for efficient CO_2_ hydrogenation by focusing on catalyst design, specifically by incorporating varying functionalities into the ligand. The fundamental strategy entails activating hydrogen molecule and enhancing the hydricity of the resulting metal-hydride species, which is based on the following two concepts of catalyst design: (i) Activating a metal-hydride by electronic effects; and (ii) accelerating H_2_ heterolysis. We have elucidated the mechanism for accelerating H_2_ heterolysis using a state-of-the-art catalyst that contains an *actor-ligand* that responds to or participates in catalysis as opposed to a classical *spectator-ligand*.

We have also demonstrated a novel heterogeneous catalysis using a molecular catalyst as a key step for the hydrogenation of CO_2_ to methanol beyond formic acid formation. The dehydrogenation of formic acid as a reverse reaction of formic acid hydrogenation is strongly favored in acidic aqueous solution. To circumvent the equilibrium limitation, we have envisioned an alternative route that both prevents the liberation of formic acid into the reaction medium, and develops a multinuclear complex to facilitate the transfer of multiple reactive hydrides. The unconventional gas–solid phase catalysis is capable of preventing the liberation of formate species and promoting further hydrogenation of formic acid through multihydride transfer.

This novel catalytic system, which is the fusion of a molecular catalyst in heterogeneous catalysis, provides high performance for methanol synthesis through a sophisticated catalyst design and straightforward separation processes. A detailed mechanistic analysis of molecular catalysts in the gas phase would lead to significant progress in the field of Surface Organometallic Chemistry (SOMC).

## Key References

- KanegaR.; OnishiN.; TanakaS.; KishimotoH.; HimedaY.Catalytic Hydrogenation of CO_2_ to methanol Using Multinuclear Iridium Complexes in a Gas–Solid Phase Reaction. J. Am. Chem. Soc.2021, 143, 1570–157633439639 10.1021/jacs.0c11927.^1^ Novel approach for methanol production by CO_2_ hydrogenation was achieved using dinuclear iridium complex under gas–solid phase conditions at lower reaction temperature.
- TsurusakiA.; MurataK.; OnishiN.; SordakisK.; LaurenczyG.; HimedaY.Investigation of Hydrogenation of Formic Acid to Methanol using H_2_ or Formic Acid as a Hydrogen Source. ACS Catal.2017, 7, 1123–1131.^2^ Hydrogenation/disproportionation of formic acid to methanol catalyzed by iridium complexes with electronically tuned ligands was investigated.
- SordakisK.; TsurusakiA.; IguchiM.; KawanamiH.; HimedaY.; LaurenczyG.Carbon dioxide to methanol: the aqueous catalytic way at room temperature. Chem. Eur. J.2016, 22, 15605–1560827582027 10.1002/chem.201603407.^3^ Direct CO_2_ transformation to methanol in a one-pot homogeneous catalysis was demonstrated in aqueous solution at lower temperature in the presence of sulfonic acid.

## 1 Introduction

The huge amount of anthropogenic CO_2_ emissions from fossil fuel combustion is undoubtedly a major contributor to climate change and should be mitigated and reduced. Considering the amount of CO_2_ emitted, technologies for converting CO_2_ to bulk chemicals or energy carriers by valorization are incredibly important. Among the possible products synthesized from CO_2_, methanol is a major commodity chemical; approximately 100 Mt is produced annually. Methanol is industrially produced from syngas (CO/H_2_) with small amounts of CO_2_ at 250–300 °C and 5–10 MPa by heterogeneous catalysis using Cu/ZnO-based catalysts. It is also a raw material for many chemicals and fuels, such as formaldehyde, light olefins, gasoline, methyl tert-butyl ether, and dimethyl ether. Therefore, it may be possible to replace most fossil-fuel-based hydrocarbons and petrochemicals with methanol derivatives.

Recently, the trend in research and development (R&D) in the synthesis of methanol has shifted toward a greener process, where CO_2_ is reduced using H_2_ produced with renewable electricity. The product is generally called e-methanol, and the process involves significantly decreased greenhouse gas emissions during the entire life cycle. However, developing the e-methanol process, which is generally performed using Cu/ZnO-based catalysts at 200–300 °C and 3–10 MPa, remains a significant challenge, because converting CO_2_ to methanol under mild reaction conditions that are compatible with varying renewable energy sources is difficult.

In this account, we disclose the technical problems associated with CO_2_ hydrogenation and provide a new catalyst design concept for CO_2_ hydrogenation to methanol under mild reaction conditions. Emphasis is placed on how flexibility in ligand design can be used to enhance catalytic performance. First, we highlight how catalyst design affects the conversion of CO_2_ to formate by the activation of H_2_ by electronic and secondary coordination sphere effects. Second, a synthetic strategy that incorporates a multinuclear catalyst under gas–solid phase conditions is explored to favor formic acid hydrogenation over the reverse reaction (i.e., formic acid dehydrogenation).

## 2 Thermodynamic Considerations for CO_2_ Hydrogenation to Methanol


**Figure 1:**
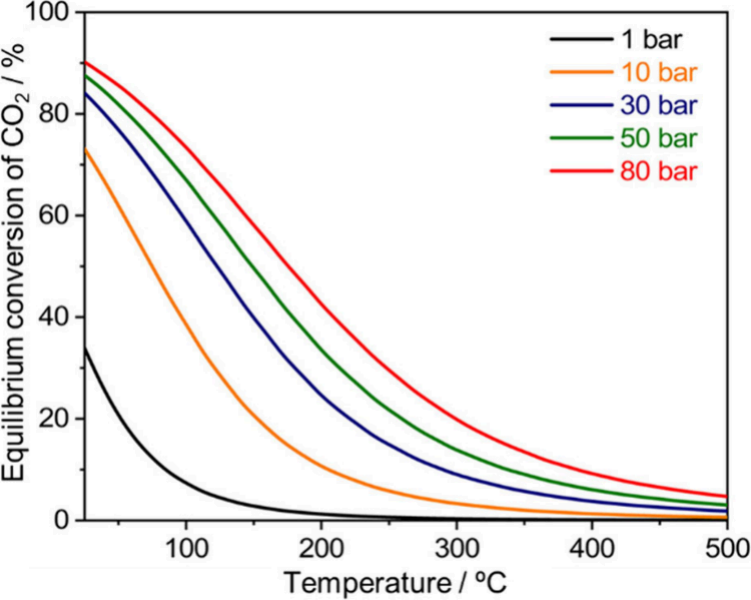
Equilibrium conversion of CO_2_ to methanol as a function of the temperature. Reprinted with permission from ref (5). Copyright 2024 Elsevier.

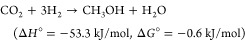
1
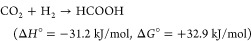
2
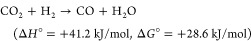
3Since CO_2_ is a final product of combustion and is thermodynamically stable, its transformation often requires harsh reaction conditions. Although the endergonic steps involved in the formation of CO and formic acid are significant barriers to the formation of methanol from CO_2_ (eqs 2 and 3), the entire reaction is an exothermic process (eq 1). Indeed, methanol formation at 4 MPa is an exergonic process at 25 °C (Δ*G*° = – 18.76 kJ/mol), whereas it is endergonic at 250 °C (Δ*G*° = + 8.98 kJ/mol).^4^ Therefore, CO_2_-to-methanol conversion is limited at elevated temperatures (Figure 1).^5^ Thus, it is necessary to develop a highly efficient and selective catalyst for hydrogenation of CO_2_ to methanol that operates at low temperatures.

## 3 Conventional Heterogenous Catalysis for CO_2_ Hydrogenation

Over the past several decades, intense research has been devoted to developing catalytic systems for methanol production from CO_2_ with high conversion and stability.^4−8^ Heterogeneous catalysis, with its compatibility with industrial processes and straightforward operation, is an effective means of achieving this goal. Among the catalysts that have been reported in the literature, Cu/ZnO-based catalysts have been most widely employed as solid catalysts under heterogeneous gas–solid phase conditions at generally 200–300 °C and 3–10 MPa. A representative example is the Carbon Recycling International plant located in Iceland, which has a production capacity of approximately 5 million liters of methanol per year using local hydrothermal and geothermal energy sources. Several commercial CO_2_-to-methanol plants utilizing catalysis have also been demonstrated in China, Chile, and the United States.

There are two accepted pathways for CO_2_-to-methanol conversion:^4−8^ (i) the CO intermediate pathway via the reverse water-gas shift reaction and subsequent hydrogenation; and (ii) the formate pathway in which CO_2_ hydrogenation proceeds through the formation of adsorbed formate intermediates. Both pathways include adsorption and activation steps of gases on the surface of the catalyst via the Eley–Rideal or Langmuir–Hinshelwood mechanisms. A comprehensive understanding of these mechanisms and catalytically active sites can guide the rational design of catalysts to improve their efficiency and mitigate side reactions. Although considerable efforts have been devoted to understanding these processes, the overall reaction pathway remains ambiguous and the nature of the active sites is still under debate.

Classical Cu/ZnO-based catalysts generally operate at temperatures above 200 °C, which limits the theoretical yield due to the exothermic nature of this reaction. In addition, the catalytic activity decreases gradually because of water-induced sintering and deactivation of the active sites. Thus, the development of solid catalysts that enable low-temperature operation has been vigorously pursued.^9−11^ However, the mechanism and limitation for the Cu/ZnO-based catalysts are beyond the scope of this account.

## 4 Homogeneous Catalysis for Methanol Production by CO_2_ Hydrogenation

In this section, recently reported molecular catalysts for methanol production by homogeneous CO_2_ hydrogenation are described. In contrast to heterogeneous catalysts, molecular homogeneous catalysts are well-defined, and a central metal stabilized by organic ligands is used as a single-atom catalyst. In homogeneous catalysis, it is relatively straightforward to obtain a better understanding of the reaction mechanism and active species using various spectroscopic techniques, which may be used to improve the stability and efficiency of the catalyst by tuning the coordinated ligand. Therefore, homogeneous catalysis can generally be performed under mild reaction conditions.

Pioneering work by Tominaga and co-workers in 1993 showed that CO_2_ can be hydrogenated to provide methanol under homogeneous conditions using a Ru_3_(CO)_12_-KI catalytic system.^12^ However, the catalysis required severe reaction conditions (240 °C and 9.14 MPa) and generated CO, methane, and ethylene as side products. Although a homogeneous system had not been reported for another 15 years, significant progress has been made recently in the field of methanol production by CO_2_ hydrogenation using molecular catalysts.^13−16^

**Figure 2:**
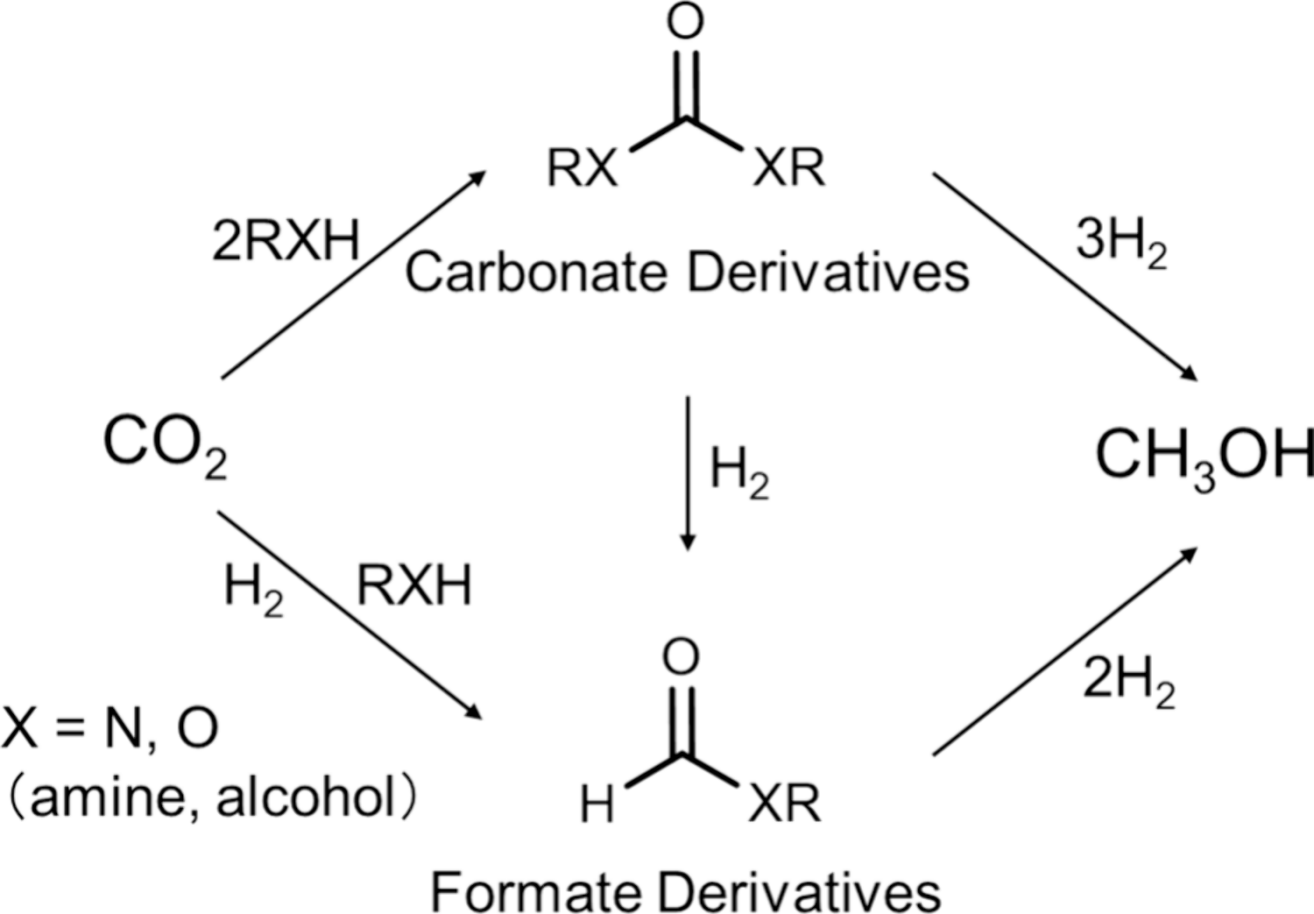
Indirect methanol synthesis by the CO_2_ hydrogenation from carbonate and formate derivatives.

The production of methanol by CO_2_ hydrogenation via carbonate or formate derivatives was explored initially.^17,18^ These indirect routes entail (i) hydrogenation of carbonate derivatives obtained by the reaction of CO_2_ with an alcohol or amine; and (ii) hydrogenation of formate derivatives obtained by CO_2_ hydrogenation and subsequent reaction with an alcohol or amine (Figure 2). As carbonates (i.e., carbonates, carbamates, and urea) and formate derivatives (i.e., esters and amides) are obtained from CO_2_ in a relatively straightforward manner, developing a catalyst for converting these substrates to methanol is essential. A variety of catalysts capable of hydrogenating carbonate precursors to methanol have been reported.^19,20^ With respect to synthesizing methanol via formamide, base-assisted CO_2_ hydrogenation has been studied extensively as CO_2_ can be hydrogenated to formate relatively easily under basic conditions. Furthermore, alkaline reagents, such as organic amines and hydroxides, have an advantage that they can efficiently capture CO_2_. This integrated approach, which combines the capture and conversion of CO_2_, is intriguing because energy-intensive CO_2_ desorption and compression steps are unnecessary.^21−24^ A strategy for methanol synthesis via formate esters by acid-assisted CO_2_ hydrogenation has also been developed.^25−30^ This catalysis sequence entails: (i) CO_2_ hydrogenation to formic acid; (ii) esterification of formic acid; and (iii) hydrogenation of the resulting ester to methanol. Lewis acids accelerate the formate ester formation.

Methanol can also be produced by the disproportionation of formic acid (eq 4), which does not require additives such as alcohols or amines.^31,32^ Formic acid plays two roles during catalysis: (i) a substrate (eq 5) and (ii) a hydrogen source (eq 6). Miller reported the first example of the homogeneous disproportionation of formic acid to methanol using a water-soluble iridium complex in the presence of an acid.^31^ However, the dehydrogenation of formic acid (eq 6) is favored over hydrogenation (eq 5), and the selectivity and yield of methanol are limited in water.
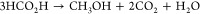
4
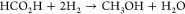
5
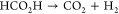
6

Direct CO_2_ hydrogenation to methanol has been achieved using Ru catalysts bearing a tridentate tris(phosphine) ligand in THF in the presence of an acid.^27,28,33^ According to analyses conducted by using nuclear magnetic resonance (NMR) and density functional theory (DFT) calculations, the hydrogenation of CO_2_ to methanol progressed without the liberation of formic acid and formaldehyde from the Ru center. An *in situ* Co catalyst with a tridentate tris(phosphine) ligand was reported by Beller and demonstrated high selectivity for producing methanol.^29,34^

Significant progress has been made in synthesizing methanol by direct CO_2_ hydrogenation under relatively mild reaction conditions by homogeneous catalysis. However, the insufficiency of catalytic activity requires additives and substrates for the formation of intermediates. Ultimately, homogeneous catalysis is inappropriate for an industrial process, owing to the inefficiency derived from complications with separating and purifying reaction solutions, including catalysts, products, and additives. More importantly, the equilibrium constraints associated with CO_2_ hydrogenation to formic acid as the first step (eq 2) should be overcome.

## 5 Catalyst Design for CO_2_ Hydrogenation to Formate

The first hurdle in transforming thermodynamically stable CO_2_ is the enhancement of catalytic activity. Pioneering work for homogeneous CO_2_ hydrogenation to formate was achieved by Inoue et al. in 1976.^35^ The significant improvement in CO_2_ hydrogenation efficiency through the use of supercritical CO_2_ was notable.^36^ However, the catalysis required harsh reaction conditions and organic additives.^37^ Joó first reported the use of complex catalysts for the hydrogenation of CO_2_ in aqueous solutions without organic additives in 1999.^38^ As a result, the current trend in research related to CO_2_ hydrogenation is toward the design of coordinated ligands as highly active and selective catalysts, which is related to their electronic properties and functionalities.

The fundamental strategy entails the activation of H_2_ and enhancing the hydricity of the resulting metal hydrides, thus facilitating hydride insertion into CO_2_, which is an important elementary step in activating CO_2_ by the formation of metal-formate intermediates. Therefore, the activation of hydrogen in catalysis is based on two design concepts: (i) activating metal hydrides by an electronic effect and (ii) accelerating H_2_ heterolysis.

**Scheme 1:**
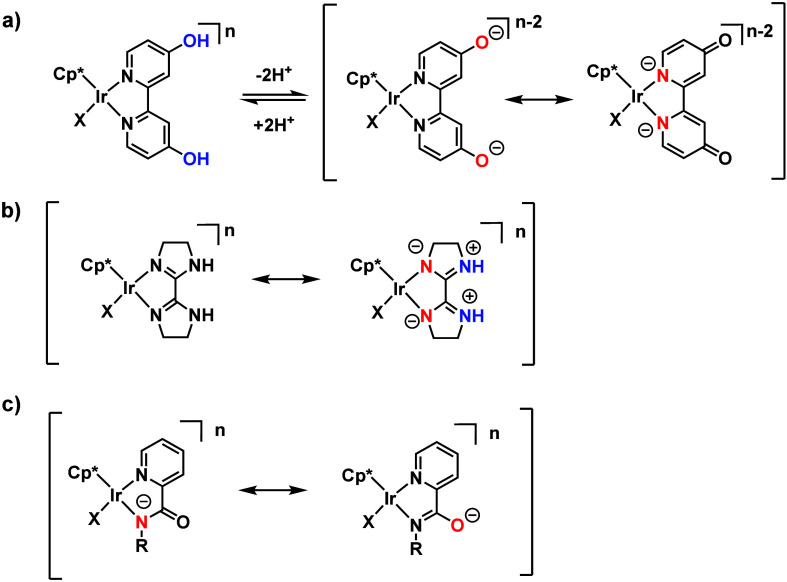
Catalyst Design Concepts for Electron-Donating Ligands in Catalytic CO_2_ Hydrogenation: (a) Proton-Responsive Ligands between Hydroxy and Oxyanion forms;^39^ (b) Localization of Electron Density;^40^ and (c) Anionic Amidate Ligands^41^

The advantage of using molecular catalysts is the possibility of fine-tuning the ligand properties, which significantly affects catalytic performance through electronic and steric effects. Furthermore, recent interest in the use of *actor ligands*, which respond or participate in catalysis, has increased relative to the use of classical *spectator ligands*, which do not respond and participate in catalysis. Therefore, we designed catalysts for the hydrogenation of CO_2_ to formate based on this strategy (Scheme 1).

**Figure 3:**
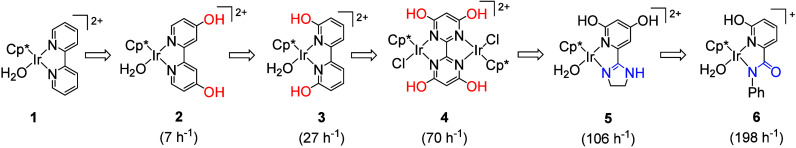
Progress of catalyst design for CO_2_ hydrogenation to formate. The values in parentheses are initial turnover frequencies (TOF) for CO_2_ hydrogenation to formate under ambient conditions.

First, we explored a prototype catalyst that functions in water. Among numerous catalysts, the half-sandwich bipyridine complexes [Cp*M(bpy)X]^n+^ (M: Rh, Ir; Cp*: pentamethylcyclopentadinyl; bpy: 2,2′-bipyridine), have been investigated extensively as catalysts for transfer hydrogenation using formic acid/formate in aqueous conditions, which is the reverse reaction for CO_2_ hydrogenation.^42^ We have also reported the transfer hydrogenation of ketones by rhodium complexes in water using formic acid as a hydrogen donor.^43^ Based on these results, we began to develop N-donor ligands for a prototype water-stable catalyst (Figure 3).

To our delight, half-sandwich iridium complex **1** produced a small amount of formate by CO_2_ hydrogenation under alkaline aqueous conditions and in the absence of any organic additives.^44^ In general, electron-donating ligands enhance the catalytic hydrogenation reactivity. Therefore, we envisioned introducing a strongly electron-donating substituent into the ligand to enhance the catalytic activity. We turned our attention toward incorporating a “*proton-responsive*” hydroxyl group into a pyridine ligand, which can be deprotonated by a base to provide a strongly electron-donating oxyanion (Scheme 1a).^45^ Surprisingly, until our report in 2004, examples of the use oxyanions in ligand were unknown in catalysis.^44^ Since the p*K*_a_ of the hydroxyl groups in **2** is 5.0 from UV–vis measurements, **2** is anticipated to exist in its oxyanion form under the reaction conditions for CO_2_ hydrogenation to formate (pH 8.3).^46^

**Figure 4:**
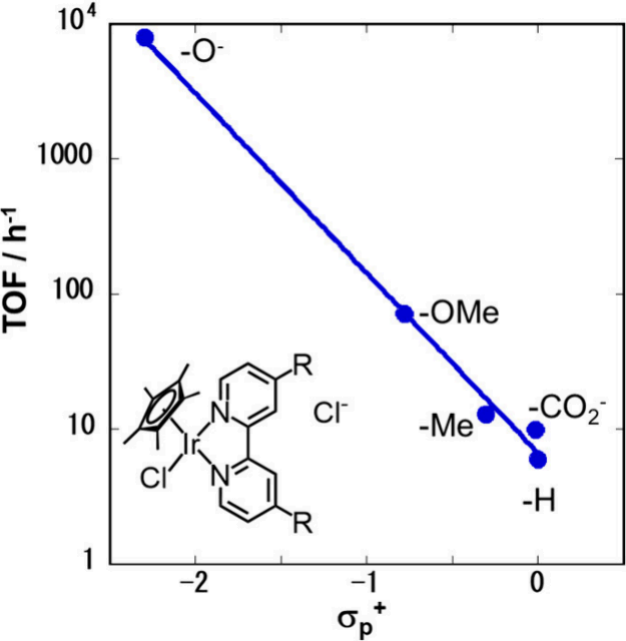
Hammett plot for CO_2_ hydrogenation to formate using [Cp*Ir(4,4′-R_2_-2,2′-bpy)Cl]Cl (R = H, CO_2_H, Me, OMe, OH) under 4 MPa of CO_2_/H_2_ (1:1) at 80 °C in 1 M KOH (aq). Reprinted with permission from ref (39). Copyright 2007 American Chemical Society.

The electronic effects of the ligand substituents on catalytic activity were examined systematically under basic aqueous conditions in a series of iridium complexes with 4,4′-disubstituted-2,2′-bipyridine (Figure 4).^39^ The results showed that the electronic effects (i.e., Hammett’s constant (σ_p_^+^)) correlated well with the observed catalytic activity (i.e., initial turnover frequency (TOF)); the initial TOF of **2** was over 1300 times higher than that of the unsubstituted analog **1** under the same catalytic conditions. Catalyst **2** can convert CO_2_/bicarbonate to formate at ambient temperature (25 °C) and atmospheric pressure of 1:1 CO_2_/H_2_ in a 1 M aqueous NaHCO_3_ solution. It is clear that the strong electron-donating oxyanion group (σ_p_^+^ = – 2.30) generated by deprotonating the hydroxyl group (σ_p_^+^ = – 0.92) enhanced the activity of **2** remarkably. Enhancing the catalytic activities in other metal complexes (Co, Ru, and Rh) using proton-responsive ligands has also been reported.^47,48^ These results indicate that electron-donating ligands activate catalysts.

**Scheme 2:**
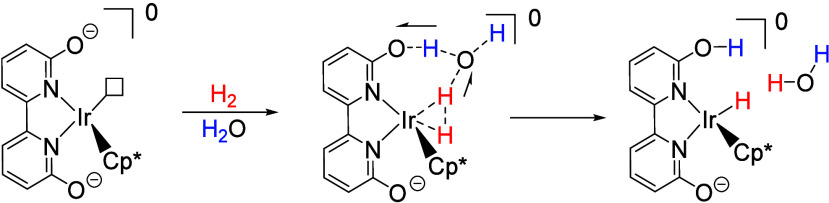
Secondary Coordination Sphere Effect: Heterolysis of H_2_ Assisted by the Pendent Base and a Water Molecule via a Proton Relay Mechanism^52^

The second catalyst design concept is to exploit secondary coordination sphere effects to reduce the energy barrier for H_2_ heterolysis.^46^ We were inspired by enzyme active sites with the use of hydrogen bonds and bases to relay protons.^49,50^ Therefore, hydroxyl groups were installed into *ortho*-positions as opposed to *para*-positions of the 2,2′-bipyridine ligand for close positioning to the metal center. Interestingly, we found the formation of iridium-hydride species to be more facile for complex **3** than for **2** in our preliminary experiments; while it took 40 h to generate 90% Ir–H from **2** under 0.5 MPa of H_2_, **3** reached 95% conversion after only 30 min under 0.2 MPa of H_2_. Furthermore, **3** showed markedly improved catalytic activity (TOF: 27 h^–1^) over **2** (7 h^–1^) for the hydrogenation of CO_2_ to formate under ambient conditions. Moreover, our experimental mechanistic studies demonstrated for the first time the involvement of water in the rate-determining step, significantly accelerating H_2_ heterolysis for **3**, which contained pendent bases near the metal center, but not for **2**.^51^ Our theoretical results, consistent with our experimental findings (i.e., kinetic isotope effects and positional effects of hydroxyl groups), indicated that the *ortho*-positioned OH-groups in **3** promoted H_2_ heterolysis through a proton relay mechanism that involved assistance from a water molecule (Scheme 2).^52^

Furthermore, the catalytic activity can be further improved by combining the individual catalyst design concepts. The tetrahydroxyl complex **4**, demonstrating the synergistic effects of **2** and **3**, exhibited significantly improved catalytic activity for CO_2_ hydrogenation and formic acid dehydrogenation.^53^ In addition, reversible H_2_ storage by combining CO_2_ hydrogenation at ambient conditions and pressurized gas release by the dehydrogenation of formic acid has been demonstrated for controlling the direction of the reaction using a “pH switch.”

For further improvement, we envisioned increasing the ligand electron-donating ability by localizing the electron density through removing its aromaticity (Scheme 1b). For example, the nonaromatic imidazoline moiety in **5** increases the electron density on the N atom by localization.^40^ More recently, we have demonstrated that amide moieties are highly effective ligands for CO_2_ hydrogenation (Scheme 1c),^41^ an important feature of which is their ability to provide strong electron-donating effects through an anionic amidate moiety and localization of electron density. By combining the electronic and secondary coordination sphere effects of oxyanions, catalysts **5** and **6** exhibited highly efficient catalytic activity for CO_2_ hydrogenation under ambient conditions.^54^ Complex **6** performed the best with a TOF of 198 h^–1^ under ambient conditions.^55^

According to our catalyst design concepts, highly effective catalysts activated by electronic and secondary coordination sphere effects facilitate CO_2_ hydrogenation under mild reaction conditions. However, the production of methanol by further hydrogenation of formate was not achieved at this stage.^56^

## 6 Methanol Production beyond Formic Acid Formation

Although several catalysts based on sophisticated ligands have achieved excellent results for the hydrogenation of CO_2_, most are blocked at the formic acid/formate stage. Therefore, the second hurdle to methanol synthesis seems to be the *endergonic* hydrogenation of formic acid to formaldehyde or methanediol, which is competitive with the *exergonic* dehydrogenation of formic acid (Δ*G*° = – 31.8 kJ/mol).^13^ As mentioned above,^13−16^ hydrogenation of CO_2_ to methanol can proceed through various derivatives, such as carbonate and formate in presence of an alcohol and amine additive, which is governed by the pH of the solution (Figure 2). However, such processes suffer from tedious separation and purification steps.

**Figure 5:**
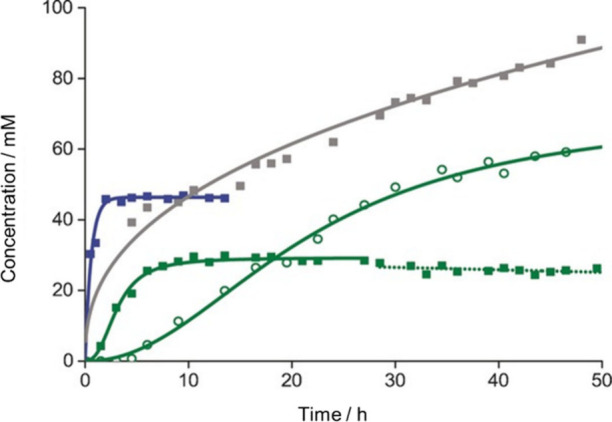
CO_2_ hydrogenation catalyzed by **2** (15.9 μmol) under 8 MPa (^13^CO_2_/H_2_; 3:1) in D_2_O. Formic acid produced at 60 °C (blue squares) and 25 °C (gray squares) without H_2_SO_4_, and formic acid (green squares) and methanol (green circles) produced in 2.5 M H_2_SO_4_ (aq.) at 70 °C. Reprinted with permission from ref (3). Copyright 2016 John Wiley & Sons, Inc.

We attempted one-pot CO_2_ hydrogenation to methanol catalyzed by proton-responsive catalyst **2** under high pressure conditions in water without any substrate.^3^ A slight amount of methanol was detected after 20 h at 50 °C and after 6 days at 25 °C in the absence of sulfuric acid. Even in the presence of sulfuric acid, little improvement in methanol production was observed. When the reaction was carried out in 2.5 M sulfuric acid aq. under 8 MPa of ^13^CO_2_/H_2_ (3:1) at 70 °C, methanol production was observed after 50 h (60 mM, TON of 7.5). Monitoring the reaction by ^13^C NMR revealed that formic acid formation preceded methanol formation, which indicates that methanol originates from formic acid (Figure 5). However, the efficiency of methanol production by the hydrogenation of formic acid is unsatisfactory, likely due to the equilibrium limitation of hydrogenation/dehydrogenation of formic acid under acidic conditions.

**Scheme 3:**
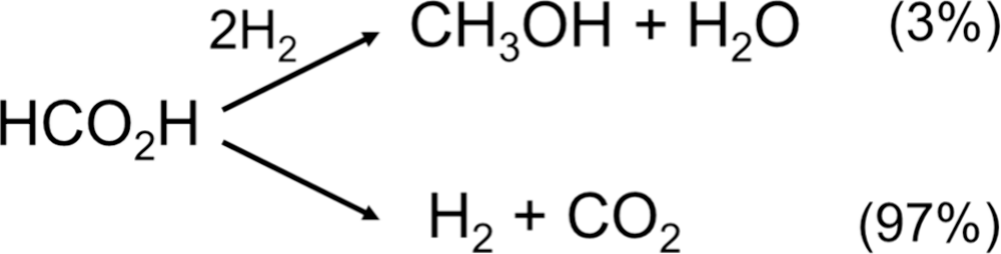
Hydrogenation vs. Dehydrogenation of Formic Acid Using [Cp*Ir(5,5′-Me_2_-2,2′-bpy)(H_2_O)](SO_4_) (5 μmol) in 4 M Aqueous Formic Acid under 3.0 MPa of CO_2_/H_2_ (1:1) at 50 °C^2^

Next, to improve methanol production, we systematically investigated the hydrogenation of formic acid by modifying the experiment previously conducted by Miller and co-workers.^2,31^ The selectivity for methanol production by formic acid hydrogenation was improved by adjusting the electron-donating effect of the various bipyridine ligand in [Cp*Ir(R_2_-2,2′-bpy)(H_2_O)]SO_4_. However, the addition of an acid was essential to produce methanol, because dehydrogenation to CO_2_/H_2_ is strongly favored in the absence of sulfuric acid. In fact, only 3% selectivity for methanol was achieved using the complex with the moderately electron-donating 5,5′-dimethyl-2,2′-bipyridine under 3.0 MPa of CO_2_/H_2_ (1:1) in the absence of sulfuric acid (Scheme 3). These results suggest that protons derived from sulfuric acid activate formic acid to facilitate its hydrogenation, and a decrease in solution pH suppresses the dehydrogenation of formic acid in the reverse direction.

**Chart 1:**
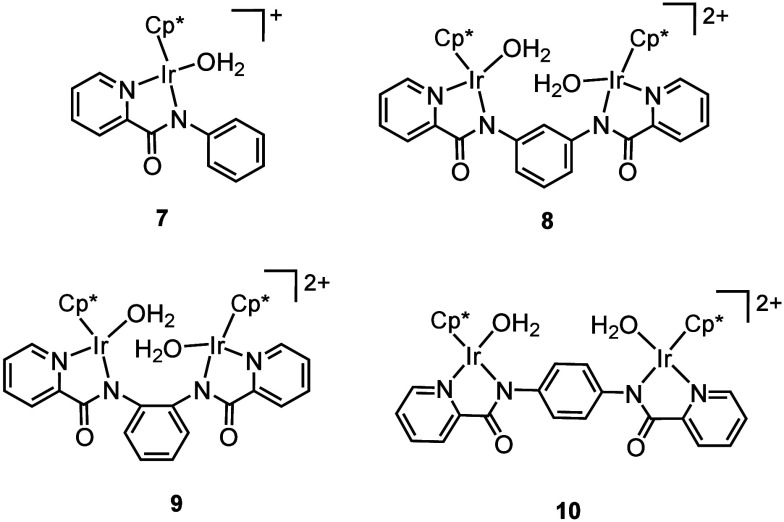
Mono- and Dinuclear Catalysts for CO_2_ Hydrogenation

Our previous studies indicated that the formation of formic acid is unfavorable for CO_2_ hydrogenation to methanol because formic acid, an initial reduction product, is easily dehydrogenated.^2,55,56^ Based on the most active catalyst for CO_2_ hydrogenation, complex **7**, having an amidate ligand, we sought to exploit a multinuclear catalyst **8** that possessed multiple active sites to achieve methanol production by concerted multiple hydride transfer to CO_2_ beyond formation of formic acid (Chart 1).^1^

**Figure 6:**
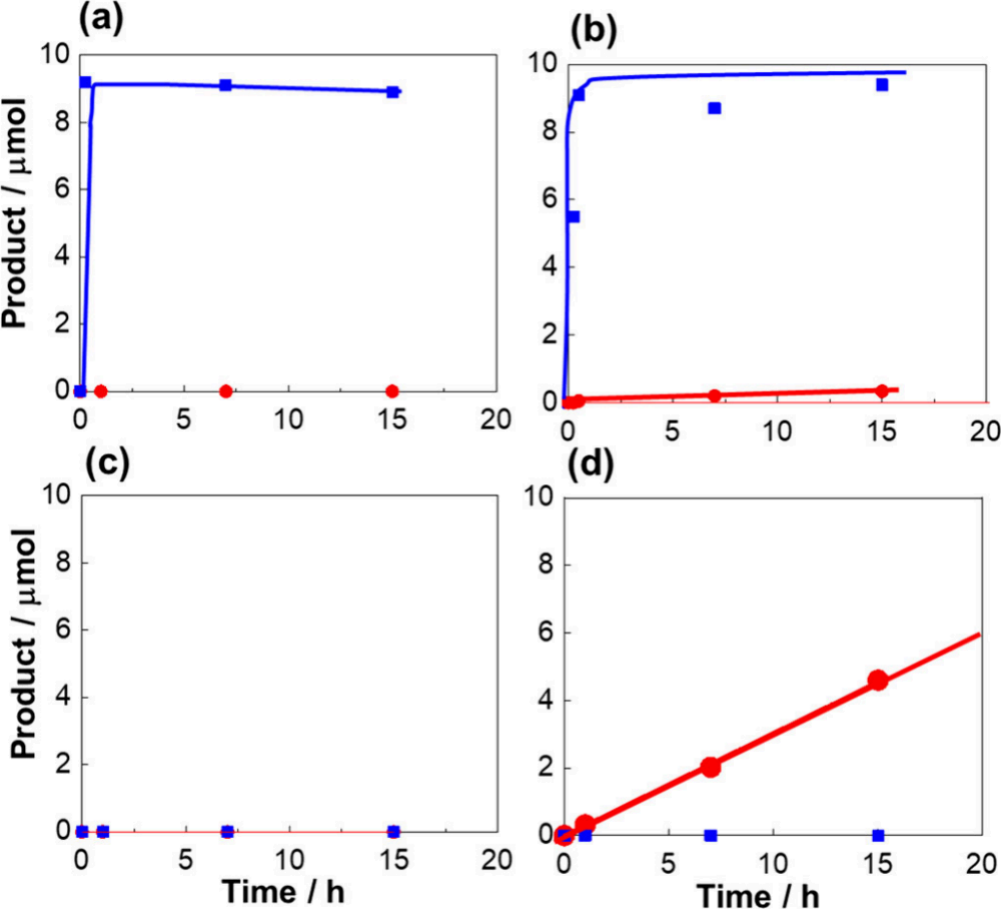
Hydrogenation of CO_2_ under 4 MPa at 60 °C catalyzed by (a) **7** and (b) **8** in water, and (c) **7** and (d) **8** under gas–solid phase conditions. Blue line = formic acid; red line = methanol.^1^

Conventional homogeneous CO_2_ hydrogenation catalyzed by mono- and dinuclear complexes **7** and **8** in water without the addition of a base was examined. Methanol formation by CO_2_ hydrogenation was not detected using mononuclear complex **7**. However, formic acid was generated immediately and saturated at equilibrium (Figure 6(a)), consistent with previous result of complex **2** (Figure 5).^3^ In the case of dinuclear catalyst **8**, although formic acid production was dominant similarly to that of **7**, a small amount of methanol was detected (Figure 6(b)). This shows the potential of **8** for methanol production via the concerted transfer of multiple hydrides to CO_2_. However, the efficiency of methanol production is unsatisfactory, owing to the liberation of formate species via ligand exchange when water is used as the solvent.

To circumvent ligand exchange with water, we envisioned an alternative route to prevent the release of formic acid and formate into the reaction medium. Thus, we attempted an unconventional gas–solid phase catalysis in the absence of a solvent.

**Figure 7:**
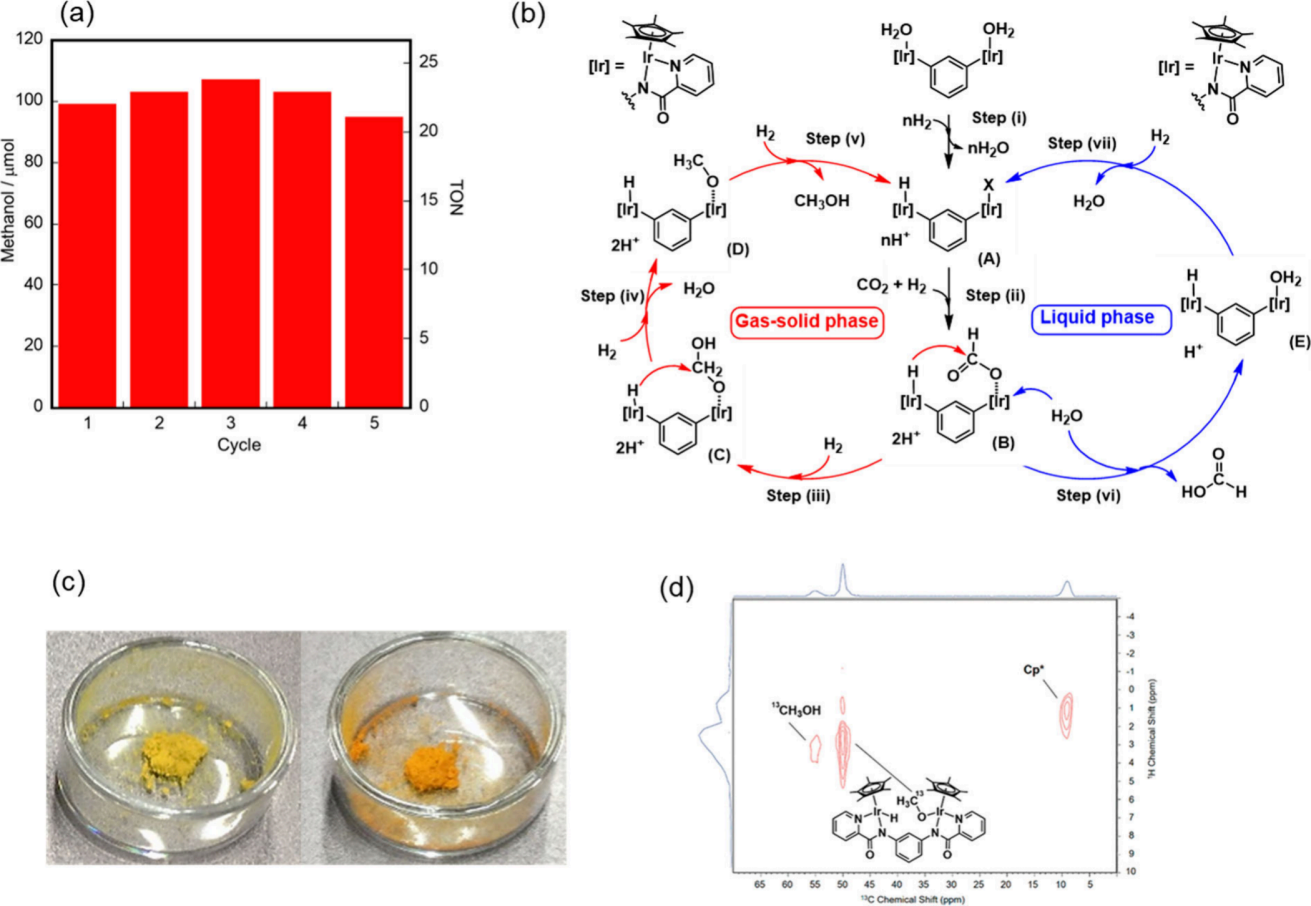
(a) Catalyst recycling experiments for the synthesis of methanol in the gas phase using **8** (4.5 μmol) under 4 MPa of H_2_/CO_2_ (3:1) at 60 °C for 336 h for each cycle. (b) Plausible mechanism for CO_2_ hydrogenation catalyzed by **8** under gas–solid and aqueous phase conditions. (c) Photographs of **8** before reaction (left) and after exposure to 4 MPa of H_2_ for 1 h (right). (d) Solid-state ^1^H–^13^C HETCOR spectrum of **8** after exposure to 4 MPa of H_2_/^13^CO_2_ (3:1). Reprinted with permission from ref (1). Copyright 2021 American Chemical Society.

The catalyst as an amorphous powder was exposed to 4 MPa of H_2_/CO_2_ (3:1) in a glass autoclave at 60 °C for 15 h. Mononuclear complex **7** did not produce methanol under the heterogeneous conditions, although a catalytic amount of formic acid was detected in the residual complex (Figure 6(c)), which implies that intermolecular hydride transfer in the mononuclear catalyst to formate species hardly occurs. To our delight, methanol (4.7 μmol) was produced exclusively with dinuclear catalyst **8** without contamination with CO and CH_4_ (Figure 6(d)). A negligible amount of formic acid was observed in the residual complex, and no formaldehyde or methanediol was detected. Even at 30 °C or 0.5 MPa of H_2_/CO_2_, methanol production was observed in the catalysis. Furthermore, heterogeneous catalysts could be recycled in a batch system by the release and refill of the reactive gas; a final TON of 113 was obtained after five cycles without any obvious degradation of the catalyst (Figure 7(a)).

The performance of the dinuclear complexes was affected by the relative configuration of the iridium centers. The *ortho*-substituted analogue **9** provided a small amount of methanol (0.2 μmol) probably due to the close distance between the iridium centers. Interestingly, in the case of the *para*-substituted analog **10**, the amount of methanol produced was dependent on its form in the solid-state. Crushed crystalline **10**_c_ formed a negligible amount of methanol (0.1 μmol), whereas amorphous **10**_a_ provided 2.6 μmol of methanol. On the other hand, amorphous **10**_ac_ obtained from **10**_c_ exhibited reactivated catalytic activity. These results indicate that intra- and intermolecular multiple hydride transfer in **10**_c_ cannot proceed because the iridium centers in **10**_c_ are too far apart, as supported by single-crystal X-ray diffraction. We believe that intermolecular multiple hydride transfer is facilitated by the π-π stacking of a suitable configuration in **10**_a_ and **10**_ac_, leading to the formation of methanol. Therefore, the relative configuration of each catalytically active site (i.e., iridium centers) is essential for the production of methanol under gas–solid phase conditions.

A plausible mechanism of hydrogenation of CO_2_ to methanol under gas–solid phase conditions is shown in Figure 7(b). In step (i), mono- or dihydride complex **A** is generated by exposure to H_2_ gas via H_2_ heterolysis. A rapid color change from yellow to orange, which can be attributed to the formation of a hydride complex, is observed upon exposure of **8** to H_2_ (Figure 7(c)). However, the transfer of protons generated through H_2_ heterolysis is of great interest because of their involvement in the formation of hydride complexes in the gas phase; the N atom of the amide moiety in the ligand may serve as a proton acceptor and facilitate H_2_ heterolysis.^41^ In step ii, the reaction of the hydride species of **A** with CO_2_ generates the formato-hydride complex **B**. In step iii, intramolecular hydride transfer proceeds from the hydride species to the formato species, generating **C**. In step (iv), further intramolecular hydrogenation of the formaldehyde equivalent in **C** affords methoxide complex **D**; the formation of a methoxy moiety is confirmed by solid-state ^1^H-^13^C HETCOR NMR spectroscopy (Figure 7(d)). Finally, the liberation of the methoxy moiety into the gas phase results in the regeneration of **A** in step (v).

In homogeneous catalysis in the liquid phase (Figure 7(b)), the formation of formato complex **B** can be presumed from the previous results under aqueous reaction conditions. However, the liberation of formic acid from formato complex **B** is speculated to occur via ligand exchange with water in step (vi). Thus, the formic acid liberated in water strongly favors dehydrogenation over hydrogenation. Consequently, only a small amount of methanol was detected in the aqueous phase.

The catalysis seems to require (1) the facile formation of a metal-hydride complex upon exposure to H_2_ in the gas phase; (2) the prevention of the liberation of formic acid from the formato complex; and (3) multiple intramolecular hydride transfers to CO_2_ by the multinuclear effect.

The synthesis of methanol by catalytic CO_2_ hydrogenation has been demonstrated using dinuclear iridium complexes under unconventional gas–solid phase conditions, which can overcome the inherent barriers of this process. This novel approach is expected to provide new possibilities for the practical production of methanol from CO_2_ using a continuous-flow reaction system under mild reaction conditions. However, a significant improvement in catalyst efficiency and a detailed mechanistic analysis of the catalytic cycle are still required.

## 7 Summary and Outlook

In this Account, we propose a novel approach for the catalytic hydrogenation of CO_2_ to methanol under mild reaction conditions. Regarding methanol production by CO_2_ hydrogenation, there are two technological barriers: the activation of CO_2_/H_2_ and an equilibrium limitation. Through our research, we have provided new concepts for catalyst design for the activation of CO_2_/H_2_ that encompass proton-responsive catalysts, secondary coordination effects, and dinuclear effects. Furthermore, gas–solid phase catalysis using molecular catalysts featuring these sophisticated catalyst designs plays an important role in overcoming the equilibrium limitation in formic acid formation. We offer novel insights into the development of methanol production by CO_2_ hydrogenation in terms of the catalyst design and reaction field. These studies will pave the way for integrating homogeneous and heterogeneous catalytic systems and their applications in practical continuous-flow reactors using molecular catalysts under mild reaction conditions. Since the heterogeneous gas–solid phase catalysis can offer simplified operating conditions with ease of separation and recycling in contrast with homogeneous liquid phase catalysis, the continuous-flow system based on our concept may be applicable to industrial processes in the future.

One of the remaining objectives pertaining to this catalytic process is the precise identification of the intermediates formed and determination of the reaction pathway. These studies would lead to the renaissance of surface organometallic chemistry,^57,58^ and we hope that further detailed investigations will aid in the rational design of future-generation catalysts.
